# Supplementary material for: Bi-MOF-Derived Carbon Wrapped Bi Nanoparticles Assembly on Flexible Graphene Paper Electrode for Electrochemical Sensing of Multiple Heavy Metal Ions
Source: Nanomaterials (Basel). 2023 Jul 14;13(14):2069. doi: 10.3390/nano13142069 (PMC10386677; doi:10.3390/nano13142069)
Supplement: Supplementary file 1 [file nanomaterials-13-02069-s001.zip › nanomaterials-2462729-supplementary.pdf]

Supplementary Materials

Bi-MOF-Derived Carbon Wrapped Bi Nanoparticles Assembly on Flexible Graphene Paper Electrode for Electrochemical Sensing of Multiple Heavy Metal Ions

Min Hu [1], Hu He [2], Fei Xiao [1,3,*] and Chen Liu [1,*]

1 Key Laboratory of Material Chemistry for Energy Conversion and Storage, Ministry of Education, School of Chemistry and Chemical Engineering, Huazhong University of Science & Technology, Wuhan 430074, China; m202170297@hust.edu.cn

2 Technology Inspection Center of Sheng Li Oil Filed, Dongying 257000, China; hehu.slyt@sinopec.com

3 Research Institution of Huazhong University of Science and Technology in Shenzhen, Shenzhen 518052, China

* Correspondence: xiaofei@hust.edu.cn (F.X.); chenliu@tjh.tjmu.edu.cn (C.L.)

Measurements and characterizations

Microstructure characterization has been performed using the scanning electron microscopy (SEM, Zeiss, Germany) and high-resolution transmission electron microscopy (HRTEM, TECNAI G220UTwin instrument, Netherlands). The elemental compositions of the samples have been analyzed using energy dispersive X-Ray spectroscopy (EDX) (EDAX Inc., Mahwah, NJ, USA). Chemical-state analysis has been performed by X-ray photoelectron spectroscopy (XPS) using a monochromatic Al-Ka (1486.71 eV) on an ESCA-LAB MKII spectrometer with a hemispherical electron energy analyzer (15 kV and 10 mA). For peak correction, all data have been standardized by the C 1s line at 284.6 eV. In addition, X-ray diffraction (XRD) has been also employed. Cycle voltammetric (CV), square wave stripping voltammetry (SWASV) and chronoamperometric experiments have been performed with the homemade potentiostat.

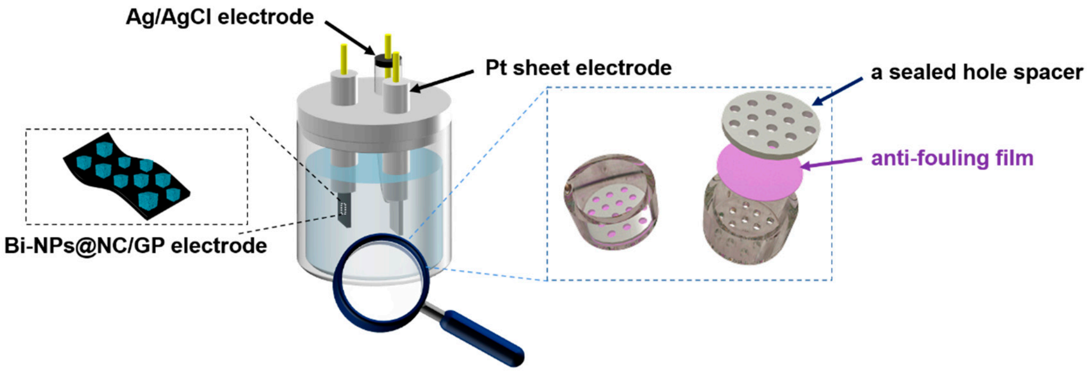

Figure S1. Schematic diagram of the structure of the three-electrode system.

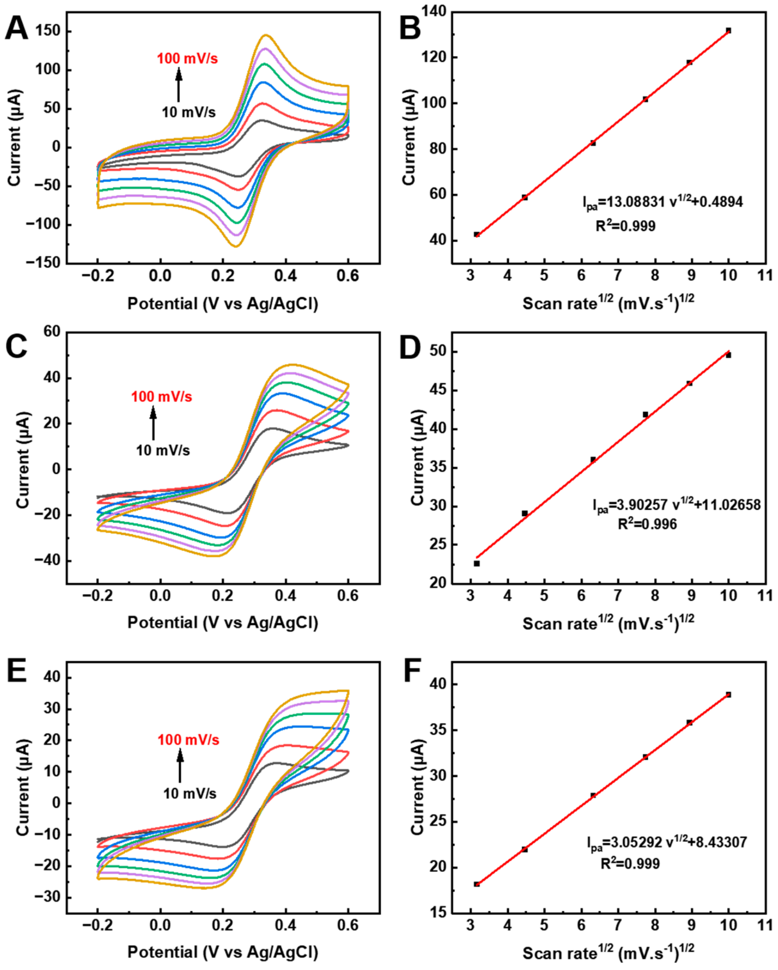

Figure S2. CV curves of (A) Bi-NPs@NC/GP, (C) Bi-NPs/GP, and (E) Bi-BDC/GOP; The linear fitting plots between the current density and scan rate of (B) Bi-NPs@NC/GP, (D) Bi-NPs/GP and (F) Bi-BDC/GOP containing 0.15 M KCl solution with 5 mM $[Fe(CN)_6]^{3-/4-}$, scan rate: 10–100 mV s^{-1}.

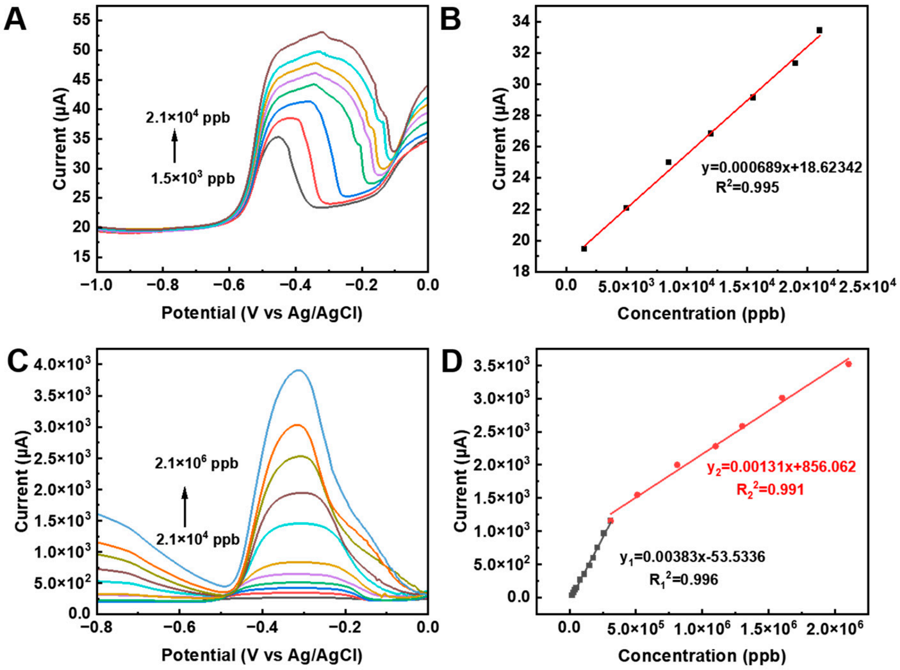

Figure S3. (A) SWASV response of the Bi-NPs@NC/GP for the individual analysis of Pb^{2+} with varying concentrations in a range of 1.5×10^3–2.1×10^4 ppb; (B) The corresponding linear calibration curves of oxidation current versus concentrations; (C) SWASV response of the Bi-NPs@NC/GP for the individual analysis of Pb^{2+} with varying concentrations in a range of 2.1×10^4–2.1×10^6 ppb; (D) The corresponding linear calibration curves of oxidation current versus concentrations. Data are presented as mean ± SD from six independent measurements.

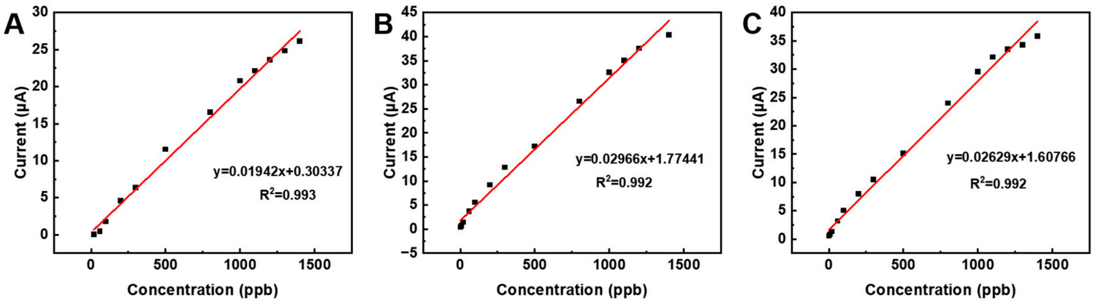

Figure S4. The linear calibration curves of oxidation current versus concentrations of (A) Zn^{2+}, (B) Cd^{2+}, and (C) Pb^{2+}. Data are presented as mean ± SD from six independent measurements.

Table S1. The linear equation parameter of Bi-NPs@NC/GP to detect simultaneous Zn^{2+}, Cd^{2+} and Pb^{2+} using SWASV under the optimal conditions.

Hybrid test	LOD (ppb)	Sensitivity (nA ppb^{-1})	Linear range (ppb)
Zn^{2+}	10	19.42	20–1400
Cd^{2+}	0.5	29.66	1–1400
Pb^{2+}	0.1	26.29	1–1400

Table S2. The analytical performances of various electrochemical sensors based on different electrocatalyst for detecting HMIs.

Electrode materials	LOD	Linear range	Ref.
Bi-NPs@NC/GP	Pb: 0.1 ppb	1–1400 ppb	This work
	Cd: 0.5 ppb	1–1400 ppb	
	Zn: 10 ppb	20–1400 ppb	
GO-Bi-NPs	Pb: 6.2 ppb	20.7–290 ppb	[1]
	Cd: 3 ppb	11.2–157 ppb	
GA-UiO-66-NH_2/GCE	Pb: 1.5 nM	0.01-4 μM	[2]
	Cd: 0.02 μM	0.06–3 μM	
UiO-66-NH_2/GaOOH	Pb: 0.028 μM	0.55–2.5 μM	[3]
	Cd: 0.016 μM	0.35–1.60 μM	
BiPs-CNFs/[EMIM][NTf_2]/CPE	Pb: 0.12 ppb	2–120 ppb	[4]
	Cd:0.25 ppb	2–120 ppb	
Modified-NPBiE	Pb:1.5 ppb	5–40 ppb	[5]
	Cd:1.3 ppb		
2D-Bi/Gra	Pb:0.3 ppb	1–30 ppb	[6]
	Cd:0.3 ppb		
BiNPs@$CoFe_2O_4$/GCE	Pb:1.51 ppb	0.06–0.6 μM	[7]
	Cd:0.92 ppb	0.08–0.8 μM	
BiNP/Nafion modified PGE	Pb:31.07 ppb	10–150 ppb	[8]
	Cd:7.31 ppb		

References

1. Bindewald, E.H.; Schibelbain, A.F.; Papi, M.A.P.; Neiva, E.G.C.; Zarbin, A.J.G.; Bergamini, M.F.; Marcolino-Júnior, L.H. Design of a New Nanocomposite between Bismuth Nanoparticles and Graphene Oxide for Development of Electrochemical Sensors. Biomater. Adv. **2017**, 79, 262-269.
2. Ru, J.; Wang, X.; Cui, X.; Wang, F.; Ji, H.; Du, X.; Lu, X. GaOOH-Modified Metal-Organic Frameworks UiO-66-NH_2: Selective and Sensitive Sensing Four Heavy-Metal Ions in Real Wastewater by Electrochemical Method. Talanta **2021**, 234, 122679.
3. Lu, M.; Deng, Y.; Luo, Y.; Lv, J.; Li, T.; Xu, J.; Chen, S.-W.; Wang, J. Graphene Aerogel-Metal-Organic Framework-Based Electrochemical Method for Simultaneous Detection of Multiple Heavy-Metal Ions. Anal. Chem. **2018**, 91, 1, 888-895.
4. Oularbi, L.; Turmine, M.; Salih, F.E.; El Rhazi, M. Ionic Liquid/Carbon Nanofibers/Bismuth Particles Novel Hybrid Nanocomposite for Voltammetric Sensing of Heavy Metals. J. Environ. Chem. Eng. **2020**, 8, 3, 103774.
5. Zidarič, T.; Hrastnik, N.I.; Šest, E.; Kovač, J.; Jovanovski, V.; Hočevar, S.B. A Novel Bismuth Imidazolate-Based Sensor for Detection of Trace Lead (II). Sens. Actuat. B Chem. **2019**, 291, 354-361.
6. Lazanas, A.C.; Tsirka, K.; Paipetis, A.S.; Prodromidis, M.I. 2D Bismuthene/Graphene Modified Electrodes for the Ultra-Sensitive Stripping Voltammetric Determination of Lead and Cadmium. Electrochim. Acta **2020**, 336, 135726.
7. He, Y.; Wang, Z.; Ma, L.; Zhou, L.; Jiang, Y.; Gao, J. Synthesis of Bismuth Nanoparticle-Loaded Cobalt Ferrite for Electrochemical Detection of Heavy Metal Ions. RAS Adv. **2020**,10, 27697-27705.
8. Palisoc, S.; Gonzales, A.J.; Pardilla, A.; Racines, L.; Natividad, M. Electrochemical Detection of Lead and Cadmium in UHT-Processed Milk Using Bismuth Nanoparticles/Nafion®-Modified Pencil Graphite Electrode. Sens. Bio-Sens. Res. **2019**, 23, 100268.
